# Prediction of nodal-line semimetals in two-dimensional black phosphorous films

**DOI:** 10.1038/s41598-020-78451-7

**Published:** 2020-12-07

**Authors:** Xiaojuan Liu, Hairui Bao, Yue Li, Zhongqin Yang

**Affiliations:** 1 State Key Laboratory of Surface Physics, Key Laboratory of Computational Physical Sciences (MOE) and Department of Physics, Fudan University, Shanghai, 200433 China; 2 Collaborative Innovation Center of Advanced Microstructures, Nanjing, 210093 China

**Keywords:** Two-dimensional materials, Condensed-matter physics, Electronic properties and materials

## Abstract

Semimetals are a new kind of quantum materials, in which the conduction and valence bands cross each other near the Fermi level. Based on density-functional theory calculations and symmetry analysis, we propose nodal-line semimetals in layered stacked black phosphorus (BP) films which are designed to have a mirror symmetry lying in the BP layer plane and thus rendering them different from the BP film systems previously studied. A closed nodal-line degenerate band can appear around the Fermi level in the BP films after a biaxial compressive strain is applied. The calculated Z_2_ number of Z_2_ =  − 1 indicates the robustness of the nodal-line semimetals obtained in the BP films, protected by the in-plane mirror symmetry. Intriguingly, with the increase of the film thickness, a smaller biaxial compressive strain is required to produce the nodal-line semimetals, more accessible in experiments. Our results provide a promising route to carrying out the nodal-line semimetals based on various two-dimensional materials.

## Introduction

Due to the unique band structures and peculiar properties, semimetals^1–15^ have drawn worldwide attention in recent years, which are generally topologically nontrivial. Topological semimetals (TSMs) can be classified into Dirac^1–3^/Weyl^4,5^, nodal-line^6–15^, and nodal-surface^15,16^ semimetals according to the dimensions (zero, one, or two, respectively) of the degenerate energy area formed by the crossing of the conduction and valence bands. The band degeneracy in the semimetals is generally protected by symmetries and is thus robust against external perturbations. The nontrivial topology of the bulk and the exotic surface electronic states in TSMs result in many intriguing quantum transport properties in TSMs, including nearly flat surface states^14^, chiral anomaly^17^, and negative magnetoresistance^17,18^. Currently, plenty of three-dimensional (3D) topological nodal-line semimetals (NLSs) are predicted in theories^6–9^, and some of them have been confirmed in experiments, such as graphene networks^6^ and Cu_3_PdN^8^. Some unique behaviors, including nondispersive Landau energy level^14^, Friedel oscillation^19^, and specific long-range Coulomb interactions^20^, have been proposed in the NLSs. For two-dimensional (2D) NLSs, the exploration is very limited in scope and depth up till now. To the best of our knowledge, some materials with honeycomb-Kagome^10^ or Lieb lattices^11^ were predicted to be 2D NLSs. Thus, comprehensive theoretical and experimental studies are indispensable for the 2D NLSs.

Black phosphorus (BP) is a layered structure and has potential applications in nanoelectronics owing to its unique behaviors like high carrier mobility, large anisotropy, and negative Poisson’s ratio etc^21–25^. 3D BPs, the most stable allotrope of phosphorus, were successfully synthesized in 1914^26^ with a layered structure. Each layer of the BP is a 2D hexagonal and puckered along the armchair direction. The specially puckered atomic structure of the BP can result in the negative Poisson’s ratio in the BP film^22^. Due to the weak interlayer interactions, monolayer or few-layer BPs can be cleaved from the 3D bulk black phosphorus. Monolayer BP flakes with the thickness of ~ 0.7 nm were exfoliated by Wang et al. in 2015^23^. Based on BP films, field-effect transistors with a high mobility of ~ 1000 cm^2^/V/s were fabricated in experiments^27^. Dirac semimetals were reported in experiments in BPs by applying an externally vertical electric field^28^. In theories, NLSs^29^ were predicted in 3D BPs by applying hydrostatic pressure etc. For 2D BP films, even though there are numerous previous researches, most of them have focused on digging out the Dirac semimetals^30,31^ and NLSs have not been explored in 2D BP films yet. For future applications, it is meaningful to explore various semimetal behaviors in 2D black phosphorus due to the need for the integration and miniaturization of the devices in nanoelectronics.

In this work, based on density function theory (DFT) calculations and symmetry analysis, we obtain NLSs in 2D layered BP ultrathin films with different thickness for the first time. For a bilayer BP built with a mirror symmetry, an elliptic closed nodal-line degenerate band is found being located around the Fermi level (E_F_) if a certain biaxial compressive strain is applied. The obtained NLS is protected by the mirror symmetry owned in the structure, confirmed by the opposite mirror eigenvalues of ± 1 for the degenerate bands. Very interestingly, the multilayer BPs, such as the four- or six-layer BPs, are also found to be 2D NLSs, under smaller biaxial compressive strains. The mechanism is analyzed in detail.

## Results and discussion

**Figure 1:**
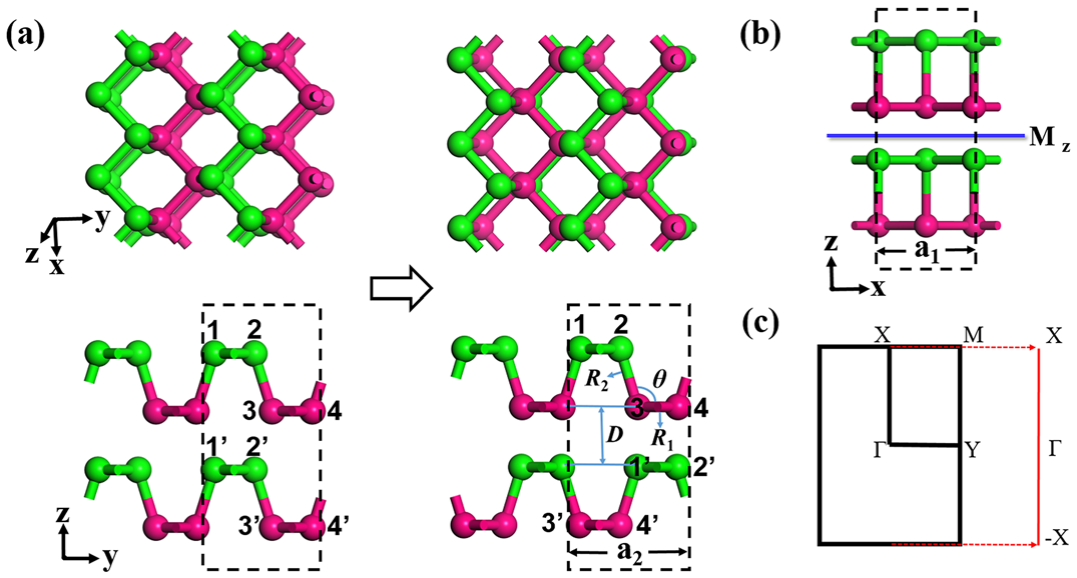
(**a**) (left panels) Top and side views of the bilayer BP, in which the top layer is stacked vertically on the bottom layer. (right panels) Top and side views of the bilayer BP we study, in which the bottom layer is shifted by 1/2 unit cell along the x and then y directions with respect to the top layer. In the built structure, the 1 and 2 atoms in the top layer are above the 3′ and 4′ atoms in the bottom layer, respectively. (**b**) The other side view (in the xz plane) of the built bilayer BP, owning an M_z_ mirror symmetry. The green and magenta colors in (**a**,**b**) indicate the top and bottom P sublattices in the nonplanar monolayer BP. (**c**) The 2D first Brillouin zone and the projected 1D Brillouin zone of the bilayer BP we build.

We focus on *N*-layer BP films (*N* = 2, 4, 6) to produce the 2D NLS. Since a mirror symmetry possibly leads to nodal-line degenerate bands^9,10^, we built a bilayer BP structure with a mirror symmetry as shown in the right panels of Fig. 1a. The weak interlayer interactions can lead to various stacking orders for the bilayer BPs, which were studied by Zhang et al.^32^ and Dai et al.^33^. The bilayer BP with the same stacking order as the 3D bulk BP has been found owning a quantum spin Hall state after certain controlling schemes are applied^32^. As shown in Fig. 1a, the bilayer BP we studied can be obtained from the bilayer BP, in which the top layer stacks vertically on the bottom layer (in the left panels of Fig. 1a), by shifting the 1 and 2 atoms of the top layer to the positions right above the 3′ and 4′ atoms of the bottom layer, respectively. The designed bilayer BP structure (right panels in Fig. 1a) has an orthorhombic lattice with a layer group 41 (space group 51, *Pmma*)^34^. The M_z_ mirror symmetry owned in the structure is illustrated in Fig. 1b. The structures of four- and six-layer BPs can be similarly built, with the same layer group and M_z_ mirror symmetry. The first Brillouin zone (BZ) and the projected one dimensional (1D) BZ of the bilayer BP film built are displayed in Fig. 1c.

**Figure 2:**
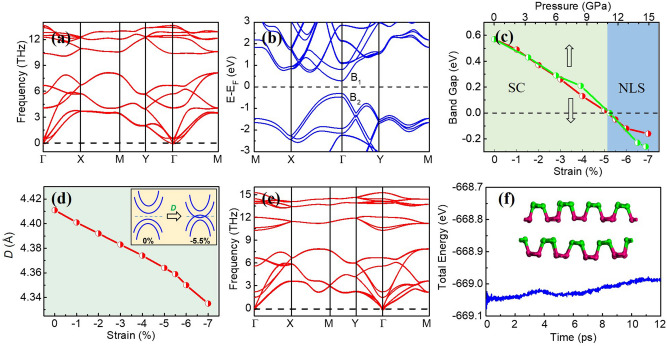
(**a**) Phonon spectrum of the pristine bilayer BP considered. (**b**) Band structure of the bilayer BP. The ‘B_1_’ and ‘B_2_’ indicate the two bands near to the E_F_. (**c**) Band gap as a function of the biaxial compressive strain (red points) and external pressure (green points) in the bilayer BP. (**d**) The interlayer distance (*D*) as a function of the biaxial compressive strain in the bilayer BP. The inset shows the evolution of the bands around the E_F_, primarily composed of the p_z_ orbitals, without and with the biaxial strain. The dash lines in the inset give the E_F_. (**e**) Phonon spectrum of the bilayer BP under a 5.5% compressive strain. (**f**) Total energy fluctuation of the bilayer BP under a 5.5% compressive strain during the MD simulation at 300 K. The inset in (**f**) shows the atomic structure of the bilayer BP at the end of the MD simulation.

The calculated structural parameters of the *N*-layer BP films (*N* = 2, 4, 6) are listed in Table 1. The parameters of the bilayer BP with a 5.5% biaxial compressive strain are also given. For the pristine bilayer BP, the optimized lattice constants are *a*_1_ = 3.298 Å, *a*_2_ = 4.628 Å. As shown in Table 1, with the increase of the layer thickness *N*, *a*_1_ increases slightly and *a*_2_ decreases gradually. *R*_1_ and *R*_2_ do not change much with the variation of the layer thickness. The interlayer distance *D*, however, increases obviously with the increase of *N*, showing the weakening of the interlayer interactions between this two BP layers*.* The applied biaxial compressive strain does not affect the symmetries the system owns. Thus, the crystal with the biaxial strain applied still possesses the mirror symmetry (M_z_). For the bilayer BP with a 5.5% compressive strain, the distance *D* between the two BP monolayers decreases much compared to that of the pristine bilayer BP, ascribed to the obvious decrease of the angle *θ* between the *R*_1_ and *R*_2_ bonds under the compressive strain. To study the structural stability of the built bilayer BP films, we construct a 4 × 4 × 1 supercell and calculate the frequency dispersion of the bilayer BP along high symmetry lines in the BZ. As shown in Fig. 2a, no imaginary frequencies appear, indicating that the bilayer BP is dynamically stable at ambient pressure.

**Table 1:** The optimized lattice constants (*a*_1_ and *a*_2_), the bond lengths (*R*_1_ and *R*_2_), the angle between the *R*_1_ and *R*_2_ bonds (*θ*), and the distance (*D*) of the layer interval having the mirror symmetry for the multiple layer BP films (*N* = 2, 4, 6).

*N*	*a*_1_ (Å)	*a*_2_ (Å)	*R*_1_ (Å)	*R*_2_ (Å)	*θ* (°)	*D* (Å)
2	3.298	4.628	2.220	2.260	104.175	4.411
2 (− 5.5%)	3.118	4.375	2.168	2.259	102.072	4.359
4	3.301	4.589	2.221	2.261	103.816	4.757
6	3.304	4.578	2.222	2.260	103.728	5.368

The second line gives the results of the bilayer BP with a 5.5% biaxial compressive strain.

The band structure of the pristine bilayer BP is presented in Fig. 2b. The band dispersions along the Г-X and Г-Y directions are so different, indicating the large anisotropy in the BP^21^. There is a direct band energy gap of 0.57 eV between the valence band maximum (VBM) and conduction band minimum (CBM) near the Г point. The band gap as a function of the biaxial compressive strain is shown in Fig. 2c. The strain is defined as (*l’ − l*) $\times$ 100% */l*, where *l’* stands for the lattice constant with a strain applied and *l* stands for the relaxed lattice constant without the strain. With the increase of the biaxial compressive strain, the band gap decreases gradually. When a 5.2% compressive strain is applied, the band gap is closed. Note that the band gap is defined as E_B1_–E_B2_, where E_B1_ and E_B2_ are the minimum and the maximum energies of the B_1_ and B_2_ bands, respectively (see Fig. 2b). Thus, the band gap becomes negative when the compressive strain is larger than 5.2%. Hence, the bilayer BP undergoes a phase transition from a semiconductor (SC) to a metal or semimetal after a compressive strain with a certain magnitude is applied. Besides the strain, applying a pressure is also an effective tuning tactics in experiments. With the extraction method mentioned in Ref.^30^, the band gap as a function of the external pressure is calculated. As displayed in Fig. 2c, when the external pressure reaches 11.2 GPa, the band gap becomes zero and the phase transition occurs.

**Figure 3:**
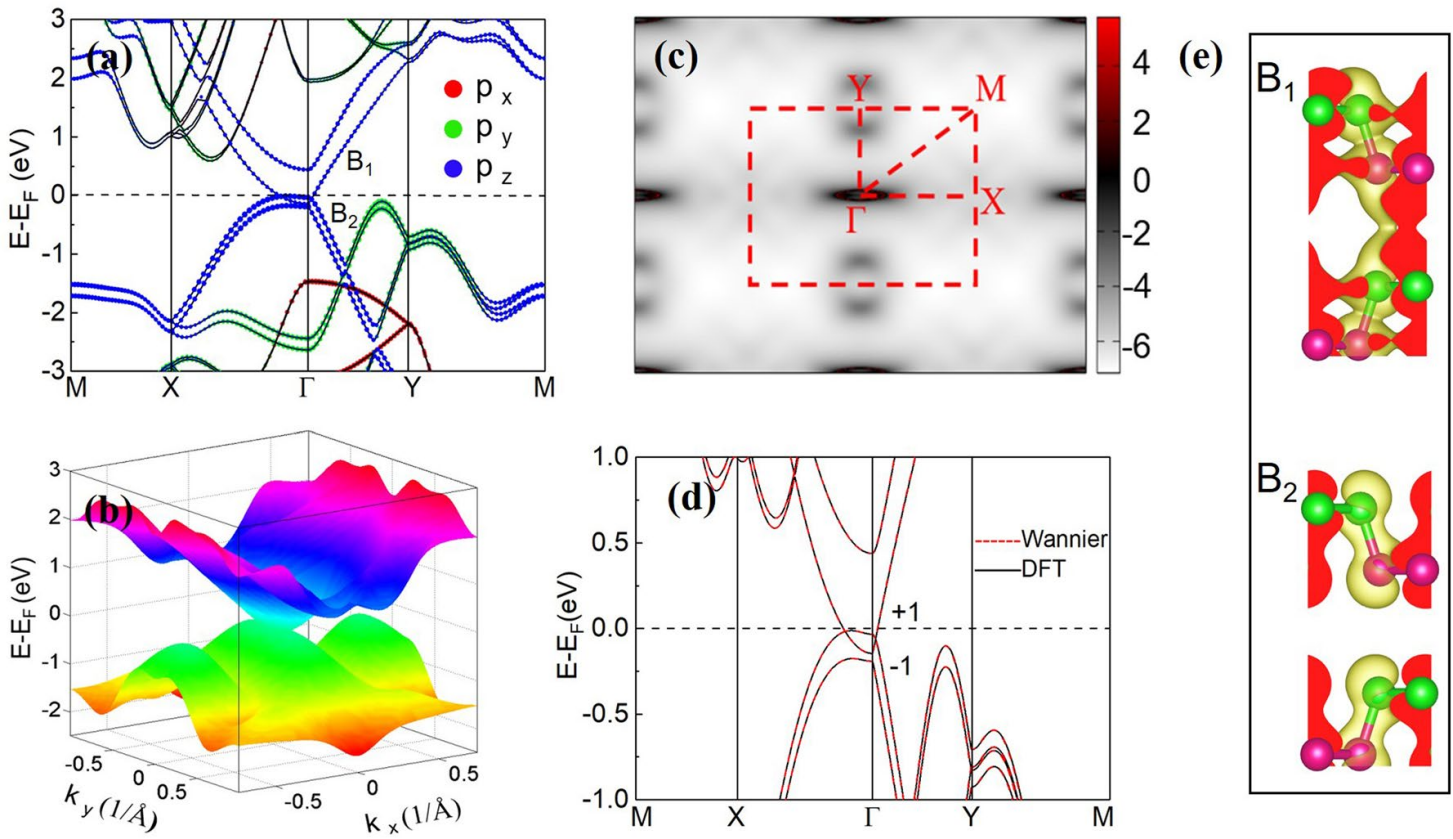
(**a**) Orbitally resolved band structure for the bilayer BP under a 5.5% compressive strain. The ‘B_1_’ and ‘B_2_’ indicate the two bands closest to the E_F_. (**b**) 3D representation of the band structure shown in (**a**), indicating a NLS formed around the Г point. (**c**) Fermi surface in the first BZ of the system. (**d**) Band structures for the bilayer BP from the DFT (black solid curves) and Wannier interpretation (red dotted curves) calculations. The bands around the E_F_ can be assigned mirror eigenvalues of ± 1. (**e**) The isosurfaces of the charge densities of the B_1_ and B_2_ bands on the yz plane, where the top and bottom P sublattices in the nonplanar monolayer are represented by green and magenta balls, respectively. The isosurface is set to be 0.01 e/Å^3^.

The mechanism of above phase transition is now analyzed. The band gap of the monolayer BP is about 1.51 eV^21^, while the bilayer BP gives a much smaller band gap (0.57 eV) (Fig. 2b), showing that the interlayer interactions between the two BP monolayers tend to reduce the band gap. For few-layer BP films, the interlayer interactions contain two aspects: the traditional van der Waals (vdW) interaction and the electronic hybridization^35^. The latter comes from the lone electron-pairs of P atoms due to the three sp^3^ hybridization orbitals formed on each P atom. This extra interaction can lead to some exceptional behaviors in the BP film, such as the thickness-dependent vibrational properties etc., not owned by graphene films^35^. Thus, the interlayer electronic hybridization and the vdW interaction together influence the electronic states when the interlayer distance *D* is varied. When a biaxial compressive strain is applied, the *D* as a function of the strain is displayed in Fig. 2d. The greater the strain strength is, the smaller the distance *D* is. Thus, the interlayer interactions are strengthened, in favor of closing the band gap. On the other hand, the bands around the E_F_ in the bilayer BP are primarily made up of the p_z_ orbitals (see Fig. 3a). Therefore, the band gap of the bilayer BP must be formed due to the bonding and anti-bonding states of the p_z_ orbitals, similar to the case in the monolayer BP^24^. As listed in Table 1, when a 5.5% compressive strain is applied, the bond length *R*_2_ decreases slightly (0.001 Å), indicating the p_z_ bond between the two P atoms (such as the 2 and 3 (or 1′ and 4′) atoms in Fig. 1a) in one BP monolayer becomes strong. This trend results in a large band gap between the bonding and anti-bonding states. The *R*_2_ change is, however, very small with the increase of the compressive strain (Table 1). Hence, the enlarging effect of the band gap from the decrease of *R*_2_ is negligible, compared to the reduction effect of the band gap from the *D* decrease in the process. Hence, the main cause of the phase transition from a SC to a metal or semimetal (Fig. 2c) is the increase of the interlayer interactions in the bilayer BP, as illustrated in the inset of Fig. 2d.

As displayed in Fig. 2c, when the compressive strain is greater than 5.2%, the band gap of the system is closed. To show the bilayer BP entering the NLS state, we take the bilayer BP with a 5.5% biaxial compressive strain as an example and analyze concretely its electronic states in following. Its structural stability is first explored. Figure 2e gives the phonon spectrum of the bilayer BP under the 5.5% compressive strain. Similar to the case without the strain (Fig. 2a), no imaginary frequencies appear in Fig. 2e, inferring the dynamical stability of the structure under the compressive strain. Besides, the thermal stability of the bilayer BP under the 5.5% compressive strain is also investigated by an ab initio molecular dynamics (MD) simulation. A 4 × 4 × 1 supercell is adopted in the calculations. As illustrated in Fig. 2f, no structural reconstruction happens and the structure remains intact at 300 K for 12 ps, indicating the thermal stability of the bilayer BP under a large compressive strain.

The orbitally resolved band structure for the bilayer BP under the 5.5% compressive strain is displayed in Fig. 3a. The result clearly reveals that a crossing of the conduction and valence bands, composed of the p_z_ orbitals, appears around the E_F_. This band inversion may lead to interesting band structures. To check whether the bilayer BP forms a NLS near the E_F_ under a 5.5% compressive strain, we plot its 3D band dispersion diagram and the corresponding Fermi surface. As illustrated in Fig. 3b, the highest occupied band and the lowest unoccupied band form a circle of band degenerate intersection near the Г point in the BZ. The Fermi surface plotted in the Fig. 3c also displays a nodal loop around the Г point. Thus, a NLS is formed in the bilayer BP with a biaxial compressive strain larger than 5.2%. We check the electronic structures of the bilayer BP film with hybrid Heyd–Scuseria–Emzerhof (HSE06) functional^36^ since the GGA functional may underestimate the band gap. The band gap obtained from the HSE06 is about 1.12 eV, larger than that (0.57 eV) from the GGA. And a larger biaxial compressive strain (> 5.2%) is expected to produce the NLS in the system. Fortunately, as illustrated in Fig. 2c, the phase transition can be induced by applying an external pressure with a not very large magnitude (11.2 GPa). Even if the HSE06 functional is employed, the critical pressure increases to 16.4 GPa, still easily carried out in experiments. Besides, as discussed below, the band gap decreases with the increase of the BP thickness. Thus, the NLS may be easily achieved in experiments in the thicker BP films. Due to the weak spin–orbit coupling (SOC) strength of the P atoms, the SOC effect is ignored in above discussion. If the SOC is considered, all the 2D crossing points do not exist anymore. Small band gaps appear along the nodal loop, consistent with the trend from the symmetry analysis that the two inverted bands have the same M_z_ eigenvalues instead of different ones. The band gaps opened along the Г-X and Г-Y directions are of 1.5 and 6.6 meV, respectively, also reflecting the large anisotropic behaviors of the BP films. Since the band gaps are very small, they are neglected in the following.

To characterize the 2D NLS obtained, we employ mirror eigenvalues of the M_z_ to define the Z_2_ number, as performed in Ref.^10^. The Z_2_ number is calculated through^10^

1 $${\text{Z}}_{{2}} = {\xi }_{ \Gamma } {\xi }_{{\text{X}}} \xi_{{\text{Y}}} \xi_{{\text{M}}} , $$

where ${\xi }_{a}=\prod_{n=1}^{{N}_{occ}}{\xi }_{an}$ (${\xi }_{a}$ is the mirror eigenvalue and N_occ_ is the number of the occupied states). For the above bilayer BP system, Z_2_ = $-1$ is obtained. Since there is only one nodal line in the BZ (Fig. 3c), we prove that Z_2_ = ($-1$)^N^_,_ where N is the total number of the nodal lines in a 2D system, i.e., N = 1 and Z_2_ =$-1$ are obtained for the bilayer BP film. Besides, as illustrated in Fig. 3d, the different mirror eigenvalues of $\pm 1$ for the two crossing bands also indicate that the degeneracy of the two bands along the nodal loop is protected by the mirror symmetry. Therefore, the formed 2D NLS in the bilayer BP is very robust, protected by the mirror symmetry M_z_. The isosurfaces of the charge densities of the B_1_ and B_2_ bands (Fig. 3a) on the yz plane are displayed in Fig. 3e. A chemical bonding-like character from the B_1_ band is obviously observed between the two P monolayers, manifesting the interlayer quasi-covalent (hybridization) interaction, which together with the traditional vdW interaction can tune effectively the band gap under the strain.

**Figure 4:**
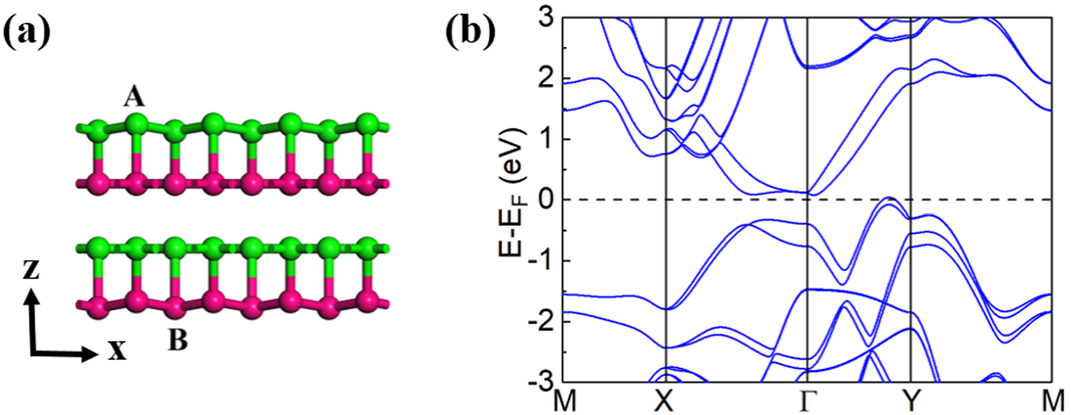
(**a**) The side view of the bilayer BP with a 5.5% compressive strain, where the M_z_ symmetry is broken by shifting the A and B atoms 0.01 and − 0.01 Å along the z axis, respectively. (**b**) Band structure of the bilayer BP shown in (**a**).

To fully understand the origin of the nodal line, we shift the A and B atoms (Fig. 4a) in the bilayer BP with a 5.5% compressive strain along the z axis by 0.01 and − 0.01 Å, respectively, to artificially break the mirror symmetry M_z_. Note that this bilayer BP without the M_z_ symmetry is less stable than that of the bilayer BP with the M_z_ symmetry (Fig. 1b) by 435 meV per unit cell. The corresponding band structure is given in Fig. 4b, from which we find that all the nodes around the Г point are annihilated and the degenerate points are all gapped after the mirror symmetry (M_z_) of the lattice is broken. An indirect band gap of 64 meV is formed in the band (Fig. 4b). This evidence shows that the nodal-line semimetal obtained in the bilayer BP with a large (> 5.2%) compressive strain is protected by the mirror symmetry M_z._ Thus, the NLSs may appear in the 2D systems if the M_z_ exists in the structures. If there is a random shift across the film and the mirror symmetry M_z_ of the structure is broken, the nodal line does not exist anymore. Due to the relatively weak interlayer interactions of the BP film, a low temperature condition is expected to observe the nodal line behavior in the BP thin film (Fig. 3a).

**Figure 5:**
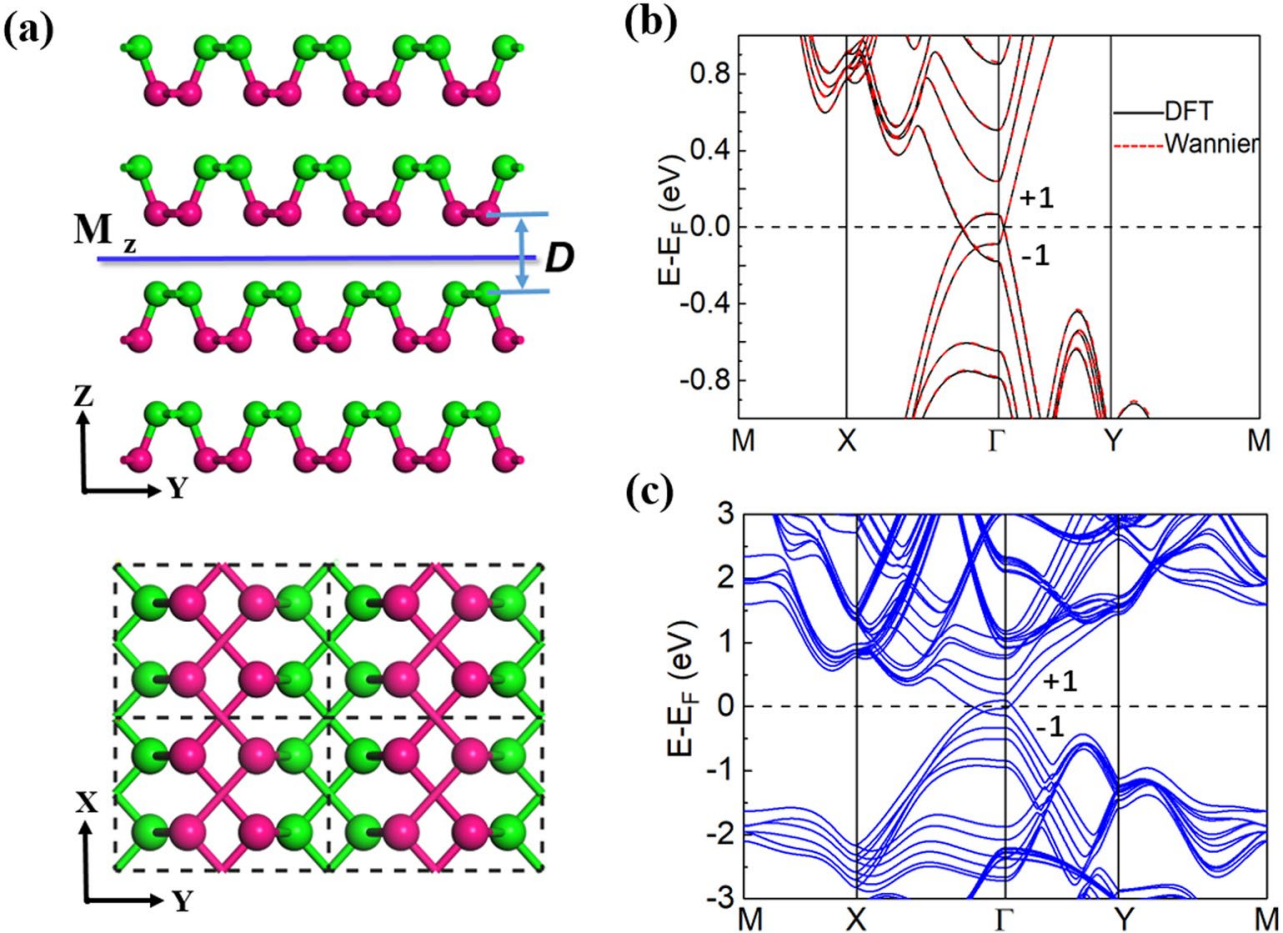
(**a**) Side and top views of the crystal structure of the 4-layer BP. (**b**) Band structures of the 4-layer BP under a 3.5% compressive strain, obtained from DFT (black solid curves) and Wannier interpretation (red dotted curves) calculations. The bands around the E_F_ can be assigned with mirror eigenvalues of ± 1. (**c**) Band structure for the 6-layer BP film under a 2.5% compressive strain. The mirror eigenvalues ± 1 are also illustrated for the bands around the E_F_.

The electronic states of the *N*-layer BP films with *N* = 4 and 6 are also explored. Since the thicker the BP film is, the stronger the interlayer interactions will be, which makes the dispersion of the valence and conduction bands larger, resulting in a smaller band gap. We, thus, expect that the thicker BP films can also possess robust NLSs as the bilayer BP, under maybe smaller biaxial compressive strains. This speculation is confirmed by our DFT results. The *N*-layer BP films (*N* = 4 and 6) also have geometrical structures with a layer group 41 *Pmma* (space group 51) and M_z_ mirror symmetry (Fig. 5a). The dispersion of the low-energy bands of the 4-layer BP film is found to be similar to that of the bilayer. The band gap becomes 0.38 eV, smaller than that (0.57 eV) of the bilayer BP. This smaller band gap of the pristine 4-layer BP leads to smaller compressive strains required to close the band gap and trigger the phase transition. Our calculations show that the band gap of the 4-layer BP can be closed with a 3% biaxial compressive strain. With the further increase of the strain, the band inversion occurs near the E_F_ and a NLS is formed in the system. The band structure of the 4-layer BP with a 3.5% biaxial compressive strain is displayed in Fig. 5b. Similar to the bilayer case, the two crossing bands around the E_F_ also have opposite mirror eigenvalues $\pm 1$ along the high symmetry lines of the Г-X and Г-Y directions and N = 1 and Z_2_ = $-1$ are obtained, indicating the robustness of the nodal-line semimetal achieved. For the 6-layer BP, the nodal line is found appearing under a biaxial compressive strain larger than 1.7% (see Fig. 5c). Thus, a NLS can be more easily acquired in the thicker BP films, favorable for experimental observation.

## Conclusion

Based on first principles calculations and symmetry analysis, we find two-dimensional nodal-line semimetals in the built multiple layer BP films with certain biaxial compressive strains applied. The nodal-line appears around the Fermi level and comes from the inversion of the two p_z_ bands with opposite mirror eigenvalues $\pm 1$. The Z_2_ = $-1$ shows the robustness of the nodal-line semimetals obtained. The thicker the BP film is, the smaller the compressive strain required to produce the phase transition from an ordinary semiconductor to a nodal-line semimetal will be. Our results provide a route to designing the two-dimensional nodal-line semimetals and promote promising applications of the black phosphorus films in future nanoelectronics.

## Methods

Our first-principles calculations are performed based on density functional theory (DFT) using the projector augmented wave method as implement in the Vienna ab initio simulation package (VASP)^37^. The Perdew–Burke–Ernzerhof generalized gradient approximation (GGA-PBE) is adopted for the exchange–correlation functional^38^. The cutoff energy is set as 400 eV for the plane-wave basis and the Brillouin zone is sampled with k meshes of 14 × 10 × 1 by using Monkhorst–Pack method^39^. Forces on the ions are calculated according to the Hellmann–Feynman theorem. The convergence threshold for the total energy is set to 1 × 10^−5^ eV. The forces on all atoms are optimized to be less than 0.01eVÅ^−1^. The phonon state calculations are carried out by using the supercell approach implemented in the PHONOPY code^40^. Molecular dynamics simulations are carried out to determine the thermal stability of the bilayer BP. The maximally localized Wannier functions (MLWFs) are constructed by employing the WANNIER90 code^41^, from which the Fermi surface is performed by WannierTools package^42,43^.
